# Can we trust projections of AMOC weakening based on climate models that cannot reproduce the past?

**DOI:** 10.1098/rsta.2022.0193

**Published:** 2023-12-11

**Authors:** Gerard D. McCarthy, Levke Caesar

**Affiliations:** ^1^ ICARUS, Department of Geography, Maynooth University, Maynooth, Ireland; ^2^ MARUM—Centre for Marine Environmental Sciences, University of Bremen,Bremen, Germany; ^3^ Institute of Environmental Physics, University of Bremen,Bremen, Germany

**Keywords:** Atlantic meridional overturning circulation, climate models, observational reconstructions

## Abstract

The Atlantic Meridional Overturning Circulation (AMOC), a crucial element of the Earth's climate system, is projected to weaken over the course of the twenty-first century which could have far reaching consequences for the occurrence of extreme weather events, regional sea level rise, monsoon regions and the marine ecosystem. The latest IPCC report puts the likelihood of such a weakening as ‘very likely’. As our confidence in future climate projections depends largely on the ability to model the past climate, we take an in-depth look at the difference in the twentieth century evolution of the AMOC based on observational data (including direct observations and various proxy data) and model data from climate model ensembles. We show that both the magnitude of the trend in the AMOC over different time periods and often even the sign of the trend differs between observations and climate model ensemble mean, with the magnitude of the trend difference becoming even greater when looking at the CMIP6 ensemble compared to CMIP5. We discuss possible reasons for this observation-model discrepancy and question what it means to have higher confidence in future projections than historical reproductions.

This article is part of a discussion meeting issue 'Atlantic overturning: new observations and challenges'.

## 1. Introduction

In August 2021, Working Group 1—The Physical Science Basis—of the Intergovernmental Panel for Climate Change published its 6th Assessment Report (IPCC AR6 WG1 [1]). With it came the reiteration that the oceans were changing due to climate change—it is ‘virtually certain’ that ocean heat content increased, and there was ‘high confidence’ that sea levels had risen. Climate models—in this case the climate models of the Coupled Model Intercomparison Project Phase 6 (CMIP6)—showed these trends would continue along trajectories governed by greenhouse gas emissions, with ‘high confidence’ that already emitted greenhouse gases will continue to warm the ocean, and it being ‘virtually certain’ that sea levels will continue to rise. However, not all icons of ocean change had such a certain affirmation—in particular, statements of confidence about observed changes in the Atlantic Meridional Overturning Circulation (AMOC) were weakened relative to the preceding IPCC Special Report on the Ocean and Cryosphere in a Changing Climate [2] in 2019.

**Figure 1.**
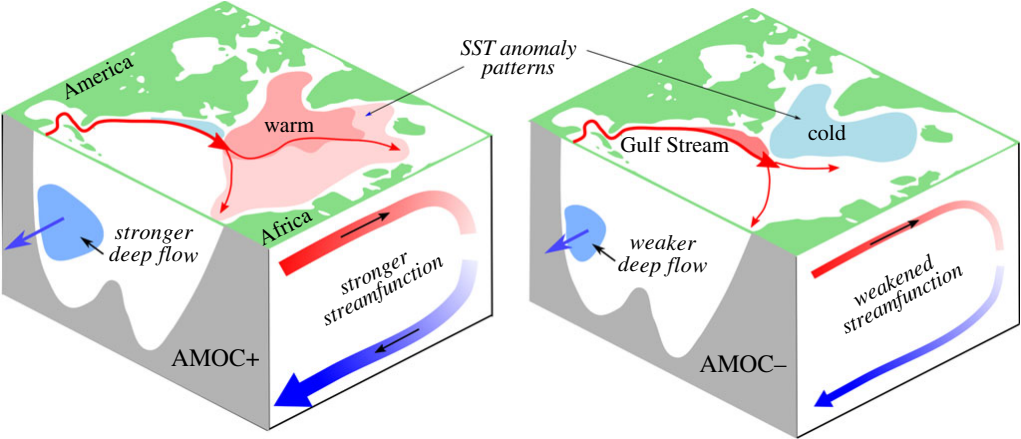
A strong AMOC (AMOC+, left) is associated with anomalously warm sea surface temperatures in the subpolar North Atlantic, more cold deep water flowing south, and a stronger overturning streamfunction. Conversely, a weak AMOC (AMOC−, right) is associated with anomalously cool sea surface temperatures in the subpolar North Atlantic, less cold deep water flowing south, and a weaker overturning streamfunction.

The AMOC is a system of ocean currents that moves warm shallow water northwards and returns cold deep water southwards (figure 1). The Gulf Stream is part of this system (hence popular use of the ‘Gulf Stream System’), but the AMOC is a much broader system of ocean circulation. The AMOC receives much attention due to its outsized role in the climate of the Northern Hemisphere and of Europe, in particular. Atlantic maritime climates in northwestern Europe are about 5°C warmer on average than Pacific maritime climates at similar latitudes [3]. The port city of Murmansk, north of the Arctic circle, remains ice free year round due to warm Atlantic waters. The popularization of ‘the Gulf Stream’ as the reason for Europe's mild climate dates from the racist [4] Matthew Fontaine Maury's *Physical Geography of the Seas* [5] and has permeated to the point that David Ellett memorably stated that in ‘the west of Scotland, chance remarks to elderly ladies upon the warmth of a winter's day invariably bring the reply “Aye, it's the Gulf Stream, you know”’ [6].

The idea that the AMOC will weaken due to climate change has a long history also. Since the mid-1980s, the earliest climate models first predicted that an AMOC slowdown would result from an increase in atmospheric CO_2_ [7,8]. This projection has been repeated in successive IPCC reports in the intervening 40 years. A large AMOC slowdown is projected to bring a cooler, stormier climate, drier summers and wetter winters, to millions of people, especially in Europe [9,10]. In the most extreme scenario of a complete AMOC collapse where no heat is delivered by the ocean to the northern North Atlantic, a cascade of climate impacts could ensue, accelerating ice loss in West Antarctica, Amazon dieback, and a collapse of arable farming in northwest Europe [11,12].

**Figure 2.**
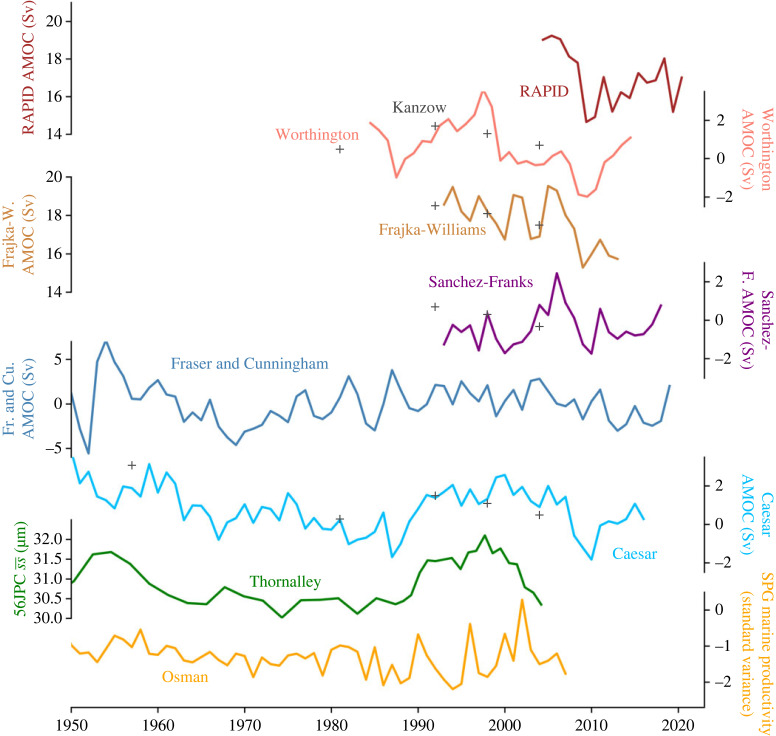
Comparison of observational AMOC data, reconstructions and proxies. Direct observational AMOC estimates from RAPID [15] are shown in brown, individual hydrographic section estimates Kanzow [16] are shown with grey crosses. Three recent reconstructions derived from RAPID at 26° N are shown: Worthington (pink) [17] based on hydrographic profiles, and Sanchez-Franks (purple) [18] and Frajka-Williams (mustard) [19] based predominantly on sea surface height. An index based on EN4 hydrographic data in the subpolar gyre Fraser and Cunningham [20] is shown in dark blue (note the larger variability of this index). The SST-based AMOC index from Caesar is shown in light blue [14] (SPG region, 6 yr lag). Finally, two palaeo-proxies that extend into the twenty-first century are shown: the sortable-silt data from Thornalley [13] (green) (DWBC, 2 yr lead, which reflects the modelled lead of the DWBC over AMOC) and the marine productivity data from Osman [21] (SPG region, 6 yr lag). The image is an updated version of fig. 2 in [22].

Whether the AMOC will weaken in the future, or perhaps has already begun to do so, is an important question. However, this question cannot be answered with certainty, which is reflected in the different assessments of the state of the AMOC as given in the IPCC reports over the last decade. While the IPCC AR5 report concluded that there was no observational evidence of a long-term AMOC decline, in 2019, the IPCC SROCC report stated with medium confidence that the AMOC had weakened relative to 1850–1900. However, by 2021, the IPCC AR6 report went back to stating there was low confidence in a twentieth century AMOC decline. How did this happen? In 2018, two independent studies were published in *Nature* that concluded that the AMOC had weakened by about 15% over the course of the twentieth century [13,14]. While the temporal evolution suggested by these proxies (Caesar and Thornalley indices in figure 2) did not fully agree with the latest climate models at that time (CMIP5) that suggested only a weak downward trend [23,24], they at least agreed on the sign of the change. Yet this changed as the latest generation of climate models (CMIP6) was released that showed, in the multi-model ensemble mean, a slight strengthening of the AMOC over the historical period [25].

In this paper, we will look at possible reasons for the model-observation discrepancy of the past AMOC evolution. First, we look at the twentieth century AMOC evolution in observational data and in the CMIP5 and CMIP6 ensembles, with a focus on the multi-model ensemble mean to mirror the approach of the IPCC. We consider how observations and models are/can be compared and discuss three possible reasons for the existing model-observation discrepancy, namely: (i) the models are wrong, (ii) the observations are wrong, (iii) models and observations estimate different quantities and are not expected to agree. Finally, we discuss what this means for our trust in model-based future projections of the AMOC.

## 2. Twentieth century AMOC in the observational data

Strictly speaking, there is not a single point in time for which a direct measurement of the full AMOC exists. Rather, the state of the AMOC is approximated by trans-basin observational systems that continuously monitor the meridional volume transport variability at different latitudes throughout the Atlantic [26]. The longest record of such a continuous directly measured AMOC time series is from the RAPID-MOCHA-WBTS programme [15], which started in 2004, and other observational programs have begun since then.

For the time period pre-2004, estimates of AMOC strength can be derived from (a) single point (snapshots) of direct, cross-basin AMOC observations from shipboard hydrography [27], (b) historical hydrographic data based on the link between changes in density of water masses and associated transports [17,20,28], (c) satellite and/or hydrographic data based on the empirical correlations between the AMOC and sea surface height variability [18,19], (d) ocean temperature or salinity observations based on the link between AMOC, and heat and freshwater transport [14,29,30], (e) paleo-proxy data that have been linked to AMOC through physical processes or in model simulations [13,31]. By design and data availability, the different types of AMOC estimates cover different time spans.

Bringing these different types of AMOC estimates together, we test whether it is possible to derive an estimate of the AMOC evolution over different parts of the twentieth century (figure 2). We focus on the evolution of the maximum of the overturning streamfunction as the most commonly used AMOC metric when comparing AMOC observations to model output. The maximum of the overturning streamfunction occurs in the subtropical North Atlantic, with the latitude of 30° N used by the IPCC.

The trends observed at RAPID consist of a strong weakening from 2004 until about 2010, following a partial recovery in recent years [32]. Going further back in time, compilations of AMOC reconstructions suggest that the AMOC weakening in the period after 2004 followed an AMOC strengthening from about 1985–2000 [33] which is also in agreement with the results of forced model runs [34]. Prior to the 1980s few instrumental reconstructions of the AMOC exist but those that do indicate the AMOC weakened from the 1960s to the 1980s [22,31], leading to an overall weakening in the latter half of the twentieth century. The problem is that due to the decadal variability seen in the AMOC reconstructions, the sign of the long-term weakening trend depends on whether the AMOC weakened before the 1980s, i.e. from a time with little observational evidence.

## 3. Twentieth century AMOC in climate models

When assessing the state of the AMOC in climate assessments, such as IPCC, typically look at the multimodel ensemble mean evolution of the AMOC, estimated by the maximum of the overturning streamfunction in the subtropical North Atlantic (that is at 26° N or 35° N). Taking the multimodel ensemble mean can reduce the uncertainties associated with individual models and simulations, since all climate models have biases [35]. It therefore provides a more robust representation of the average response of the climate system to a given scenario or forcing.

The largest framework to compare and evaluate climate models is the Climate Model Intercomparison Project which began in 1995 and is currently in its sixth phase. We look at the evolution of the AMOC at 26.5° N in historical simulations from both CMIP5 and CMIP6. As the AMOC streamfunction was not uploaded to the CMIP archives we make use of the streamfunction calculations of Menary *et al.* [36]. We therefore use the same pool of models and ensemble members as them, which are 41 models (totalling 146 members) from CMIP5 and 27 models (totalling 135 members) from CMIP6 (see electronic supplementary material, table S1). As the historical simulations end in 2014 (2004), the CMIP6 (CMIP5) data is extended with the scenario data from SSP585 (RCP8.5). This limits the number of available simulations to 84 (CMIP5) and 56 (CMIP6). To avail ourselves of as much data as possible, we calculate all trends for the pre-2004 period (this is when the historical CMIP5 simulations end) from the large ensemble and only limit ourselves to the smaller one when it is necessary, i.e. when the post-2004 data are needed. This is noted in the text accordingly.

**Figure 3.**
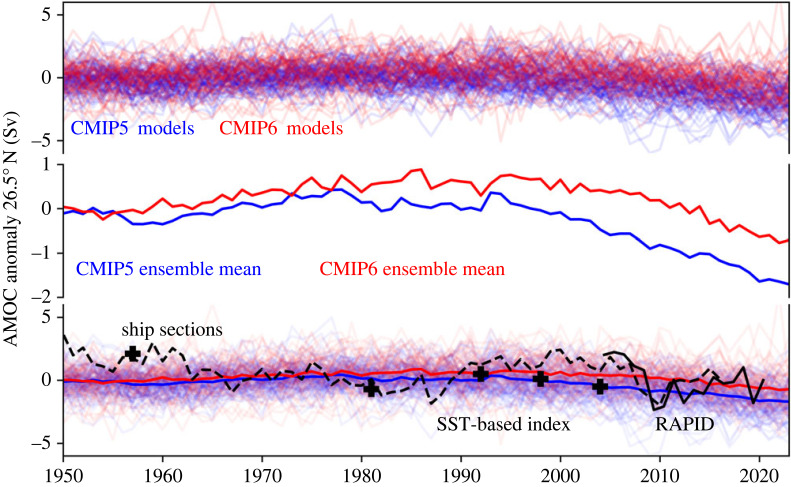
Evolution of the AMOC in the historical simulations of the CMIP5 (blue) and CMIP6 (red) ensembles (extended with the RCP8.5 and SSP585 scenario, respectively; therefore, only the smaller pool of ensemble members is plotted) compared to observational data (black). The upper panel shows the spread of the ensemble members, the middle panel shows the evolution of the multi-model ensemble mean and the lower panel shows both compared to the observational RAPID data (solid black line), the SST-based AMOC index (dashed black line) and the observational single point AMOC estimates based on hydrographical ship sections (black crosses).

While the spread of the ensemble members of each model about the ensemble mean of each model is roughly similar between the CMIP5 and CMIP6 models (figure 3, upper panel), the historical evolution of the ensemble means shows some pronounced differences. The CMIP5 ensemble mean is fairly stable before starting to decline around the year 2000, the CMIP6 ensemble mean actually strengthens until approximately 1985 and then starts to decline afterwards (figure 3, middle panel). The differences between the two generations can be better identified when looking at the linear trends over different time periods (table 1).

**Table 1.** Comparison of the linear trends (given in Sv/dec) found in the historical evolution of the AMOC in the CMIP5 and CMP6 model ensembles for different time periods. Linear trends were calculated for each model and ensemble member individually, giving the ensemble distribution of linear trends. Given is the mean of the ensemble distribution ± 1 s.d. Trends were calculated based on the full pool of ensemble members (146 for CMIP5 and 135 for CMIP6), unless the time period is denoted with an asterisk (*), then only the reduced pool of 84 (CMIP5) and 56 (CMP6) members is considered.

	1900–2000	1950–2020*	1950–1985	1985–2000	2000–2020*
	[Sv/dec]	[Sv/dec]	[Sv/dec]	[Sv/dec]	[Sv/dec]
CMIP5	−0.04 ± 0.13	−0.14 ± 0.20	0.10 ± 0.33	−0.11 ± 0.92	−0.67 ± 0.58
CMIP6	0.07 ± 0.21	−0.00 ± 0.29	0.27 ± 0.43	−0.02 ± 1.00	−0.59 ± 0.67

While the CMIP5 models on average suggested a slowdown of the AMOC over the twentieth century [24], the CMIP6 models indicate an increase in AMOC strength [37]. Looking at the different time periods in detail, we can see that this difference mainly stems from the fact that the CMIP6 models simulate a fairly strong increase in AMOC strength from 1950 to 1985, where the CMIP5 models only simulate a slight increase. Studies found that these differences are likely due to an on average stronger anthropogenic aerosol forcing in CMIP6 than CMIP5 [36,37]. They also suggested, based on observational constraints, that the anthropogenic forcing and/or the AMOC response in the CMIP6 models may be overestimated.

After approximately 1985, the AMOC started to decline in both the CMIP5 and CMIP6 models with an increased slowdown after the year 2000. While the overall post-1950 trend is on average negative for the CMIP5 models, it is basically zero for the CMIP6 models. The larger standard deviation of the CMIP6 trends furthermore shows that the spread within these models is larger than the one within the CMIP5 models (table 1). This could be seen as somewhat surprising as in general, the CMIP6 models due to, for example, their higher spatial resolution and improved representation of clouds and aerosols, are an improvement over the CMIP5 models which might lead to expectations of a convergence in AMOC trends and lower standard deviation. Yet an improved representation of these processes does not necessarily lead to a convergence. Indeed, there are other climate parameters whose range have increased from CMIP5 to CMIP6 such as climate sensitivity (the temperature response of the climate system to increases in CO_2_ concentrations) [38]. Another possibility is that these differences stem from the fact that different models were considered for the CMIP5 and CMIP6 project, i.e. we did not limit ourselves to only consider those models that participated (and whose data were available to us) in both CMP5 and CMIP6. In a study by Gong *et al.* [39], this was done, i.e. they compared the AMOC in 18 models that exist in both CMIP5 and CMIP6 (albeit with different model versions including changes in the resolution). But they also found a large spread in long-term AMOC trends in the CMIP6 models, and a greater deviation of the CMIP6 models (compared to the CMIP5 models) from the mean of the RAPID observations, supporting our findings.

## 4. Comparison of the AMOC in observations and models

**Figure 4.**
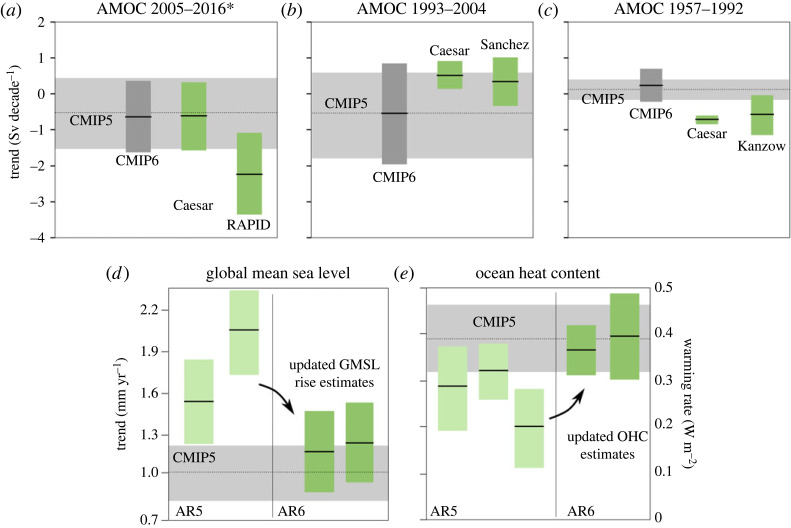
(*a–c*) AMOC trends for different periods showing the discrepancy in AMOC trends between model estimates from CMIP5 (light grey) and CMIP6 (dark grey), and observations (green). Observation labels Caesar, RAPID, Sanchez and Kanzow refer to references [14–16,18], respectively. For models, the height of the coloured area is the mean of the ensemble distribution ±1 s.d. For observations, the coloured area is the uncertainty from the linear trend regression ±1 s.d. (*d*) GMSL trends showing the reconciliation of model estimates (light grey) following improved observational estimates from AR5 (light green) to AR6 (green). Figure derived from fig. 3 of [40]. (*e*) The same as (*d*) for ocean heat content (OHC). Adapted from [41].

Comparing the models' AMOC evolution to the observational data (figure 3, lower panel), we find that neither the CMIP5 nor the CMIP6 ensemble mean are successful at representing the observational AMOC data. Looking at the only available direct continuous AMOC observations (RAPID data) we can see that the ranges of uncertainty of the 2005–2016 RAPID trend and the standard deviation of the CMIP5 and CMIP6 ensemble distributions of the same trend barely overlap (figure 4*a*). The fit between model output and SST-based AMOC index [14] for the same time period is better (figure 4*a*). However, the ability of temperature-based indices to represent AMOC strength has been challenged on certain timescales [42]. We nevertheless include this index in our consideration as it is an AMOC indicator with an annual resolution that covers the complete 1950–2020 period. Also, temperatures in the subpolar North Atlantic are dynamically linked to changes in AMOC strength on intra annual to decadal time scales [29,43,44] and the overall fit between SST-based index and RAPID observations is quite good (figure 2). For the pre-2004 period, we furthermore compare the models’ trend to a recent satellite-based reconstruction [18]: while the 1993–2004 trend of SST-based AMOC index and satellite-based reconstruction compare very well to each other and suggest a trend of about 0.5 Sv/decade, the CMIP models actually suggest a slight negative trend within the same period, albeit with a very large range of possible trends (figure 4*b*). Going further back in time (i.e. pre-1993), observational evidence becomes even more sparse. The only direct observational evidence comes from five hydrographic sections taken in the years 1957, 1981, 1992, 1998 and 2004 [27]. We therefore compare the 1957–1992 from these (for the annual cycle of the AMOC corrected) sections [16] to the trend of the SST-based AMOC index and the ones in the CMIP models (figure 4*c*). While the two observational-based trends are very similar and suggest an AMOC weakening of nearly 1 Sv/decade over this time period, the models show overall a slight AMOC strengthening during this time.

Overall, we find that the various AMOC reconstructions based on observational data have discrepancies but agree with each other better than with the multi-model ensemble means. While, for the post-2004 period the sign of the trend suggested by the CMIP ensemble means agrees with the one found in the observational evidence, it is off for the earlier time periods with the CMIP models suggesting an overall strengthening from 1957 to 1992 but a weakening from 1993 to 2004 and the observational data suggesting the opposite. It is also interesting to note that the CMIP6 ensemble mean tends to show greater discrepancies with the observations than the CMIP5 ensemble mean.

## 5. Possible reasons for the model-observation discrepancy

But why do we see such a discrepancy between model and observational data? We consider three possible reasons:

(i) The models are wrong.
(ii) The observations are wrong.
(iii) Models and observations are not expected to agree.

### (a) The models are wrong

It is a tautology that *all models are wrong*. However, the utility of climate models is that they accurately simulate elements of the climate system. Failure to achieve this can have a number of origins. For example, certain key processes may not be simulated. One problem with the current generation of climate models is that most do not include a dynamic Greenland Ice Sheet, i.e. they are not capable of modelling the increasing meltwater runoff which could drive an AMOC weakening. Other Arctic freshwater dynamics, such as the buildup of freshwater in the Beaufort gyre, have an important role in North Atlantic climate and are also not simulated by climate models [45]. Another problem is that it was shown that the historical AMOC strengthening of the CMIP5 and CMIP6 models is forced by anthropogenic aerosols [36] yet an in-depth analysis suggests that these models likely overestimate the response of the AMOC to these aerosols [37].

Models can, by definition, be tuned to fit data. In the case of complex coupled climate models, the number of fitted parameters (e.g. global mean surface temperature) are always limited. Emerging observational constraints are an effective way of constraining multiple ensembles of climate models and are a technique that has been employed in the IPCC AR6 report for the quantities of ocean heat content and sea level. For the AMOC, Bonnet *et al.* [46] employ a constraint on sea surface temperature to a climate model and examine resulting AMOC trends. This results in a declining AMOC trend from 1950 to 1970 that qualitatively (but not quantitatively) agrees with the observations during this period. An implication of this study was that the mid-twentieth century AMOC decline muted global warming—a trend that will reverse in future making it more difficult to avoid crossing the 2°C threshold.

### (b) The observations are wrong

It is possible that the problem does not lie with the models, but with the observations. The improvement of observational estimates of key climate indices leading to a reconciliation with climate model estimates has precedent. Figure 4*d–e* shows the global mean sea level (GMSL) rise and ocean heat content model-observation comparison. Disagreement has been resolved between AR5 and AR6 due to improvements to the observational estimates. In the field of sea level rise, AR5 era estimates of GMSL rise in the period 1902–1990 were consistently in the range of 1.5–2 mm yr^−1^ [47,48]. These estimates were improved methodologically using fingerprinting [40] and probabilistic methods [49], reducing the estimated rate of GMSL rise. Estimates now fall within the range projected by CMIP5 models (figure 4*d*). Similarly, in the field of ocean heat content change, AR5 era estimates of ocean warming were consistently lower than that estimated by climate models. Revised with improvements related to known biases in a type of instrument known as an expendable bathythermograph, ocean warming estimates now fall within the range projected by CMIP5 models [41] (figure 4*e*). Analogy is certainly not proof, but we present these analogies here as an appeal for continued improvements in AMOC observational estimates and reconstructions.

### (c) Models and observations are not expected to agree

It is also possible that climate models should not be expected to fully agree with observations in the historical period: the multi-model ensemble mean aims to represent the overall forced response of a system, not its internal variability (as this is cancelled out when averaging over enough ensemble members). The true internal variability (i.e. that of the real-world climate system) may only be picked up by a few individual simulations or by none at all. Bonnet *et al.* [46] found that those model runs that fit the evolution of global mean temperature most accurately showed a declining AMOC over the twentieth century. This suggests that the internal variability of the AMOC could have muted global warming over the last decades—a trend that will reverse in future, making it more difficult to avoid crossing the 2°C threshold. The results of Bonnet *et al.* [46] stem from only one model and the IPCC AR6 report indicates that most climate models fail to correctly model the larger internal variability in the observed AMOC.

When considering the multi-model ensemble mean here, we are not only mixing different ensemble members but also different models. This means that different models may respond to different external forcing, i.e. internal variability need not be the only reason for differences in the simulation. This is well shown by the differences between the CMIP5 and CMIP6 model ensemble means. Even when multiple models are considered, the response to external forcing can still be different. Therefore, we cannot say that the difference between the reconstructions and the model ensemble mean can be narrowed down to internal variability but we can consider the situation where the difference might be predominantly driven by internal variability.

If internal variability dominates AMOC evolution in the historical period, is there any hope for its simulation? We do not expect climate models forced by historical emissions to reproduce the weather or, for example, individual storms over the historical period—these simulations are not considered as weather forecasts. If the AMOC is more like a storm, then it should be forecast like a storm using initialized models. This is the approach of decadal climate prediction where climate models are initialized from observations. This approach has also proved successful in simulating decadal Atlantic climate variability with the Atlantic warming in the 1990s and cooling in the 1960s successfully reproduced using this approach [50,51]. Indeed, AMOC hindcasts from decadal prediction systems show similar decadal evolution to the converging lines of evidence from the observations: weakening in the 1960s and strengthening in the 1990s [52]. While decadal predictions may offer a successful way of modelling AMOC evolution, their success could be evidence of large internal variability, and consequently pointing to the unlikelihood of historical climate models reproducing AMOC variability.

There also lies the possibility that both models and observations are wrong. It could be argued that if we accept that *all models are wrong* as a tautology, then we should accept that all observations are wrong also. This is at least true in the case of the leading AMOC metric—that of the maximum of the overturning streamfunction. For example, consider the leading observational estimate from the RAPID programme. This metric for AMOC evolution explicitly includes Ekman transport. Ekman transport is ageostrophic, adjusts over short timescales and is driven by local winds. Ekman transport does not naturally sit in a conceptual framework for the AMOC such as presented in the schematic in figure 1 but it has had important impacts on the RAPID metric for AMOC strength [53,54]. Also, we know that the overturning streamfunction varies on differing timescales in the subpolar and subtropical gyres [55] so which gyre provides a better estimate of the AMOC? Is there a better AMOC metric that would unlock the known coherence [56] across gyres?

## 6. Discussion

If the observations of the AMOC are wrong and the multi-model mean of the CMIP6 ensemble members does represent the twentieth century AMOC, the only problem with the models is that there is a large range of trends in the CMIP6 ensembles. Ultimately, if external forcing of the AMOC is of leading importance, then the key variations in the multi-model mean of the CMIP6 ensemble members should capture the evolution of the AMOC in the twentieth century. Moreover, this multi-model ensemble mean has remained relatively consistent between CMIP5 and CMIP6, with changes to the magnitude of AMOC change rather than the sign of that change. The type of external forcing that does dominate the AMOC in this scenario is a relevant concern. Future AMOC simulations differ little between greenhouse gas trajectories until beyond 2050 when aerosol forcing fades out. Aerosol forcing has been key to historical changes in AMOC in CMIP models [36]. By contrast, there has been little discussion of the role of aerosols in observational AMOC literature. Could aerosol forcing of AMOC be key and the current set of AMOC observational reconstructions be based on incorrect model relationships?

Were aerosol forcing to be key, it offers another path of reconciliation between observations and climate models. Many observational AMOC proxies depend on the relationship between subpolar warming and a strong AMOC [14,57]. However, this is not the only potential relationship. In certain models, where aerosol forcing dominates, an inverse relationship between subpolar warming and AMOC strength is found. Potentially, incorrect relationships that underestimate aerosol forcing of AMOC in observations could be the cause of the disagreement. This statement can be applied more generally as many of the observational proxies are reliant on relationships derived from models and therefore reliant on those models getting this relationship correct. Some of the sparse hydrographic AMOC estimates that currently disagree could be explained by aliasing large natural variability. Progress on this can be made by further understanding what historical hydrography is telling us about AMOC variability.

Potentially, we do not expect the historical climate models and the observations to match. Internal variability may dominate observations. The multi-model ensemble spread only barely encompasses the range of AMOC observations as shown in figure 3. This suggests that natural internal variability is very large and, while it does not exclude the possibility that certain model ensemble members capture the correct size of internal variability, suggests that it may be the limit of the models' ability to capture it. In this case, we do not expect the climate models to match observations, but can we explain the magnitude of internal variability and what are the implications for the future?

If internal AMOC variability is that big, then future climate could be incorrectly projected because forced models are being tuned to internal variability. AMOC variability may have masked the global warming trend [46]. Likewise, if AMOC variability is all (or at least mainly) internal and multidecadal, the AMOC change could mask other changes in future climate. Progress could be made in this scenario by the use of observational constraints to pre-select ensemble members on the basis of history matching. All models and ensemble members are not equal. The use of observational constraints has been integrated into the IPCC process for other variables such as sea level and could also be applied to AMOC. An alternative would be to stretch the limits of decadal predictions to truly decadal timescales of 30 years or so, much longer than current standards. All of these scenarios have important implications for the climate sensitivity—such a hot topic for CMIP6 models in particular. Could accurate representation of AMOC in climate models reshape our view on future climate change?

Of course, it could be that the models are wrong. They are missing important physical processes such as cryospheric, freshwater and deepwater formation processes. The impact of resolution (ocean and atmospheric grids) for air–sea interactions and their link with the AMOC needs to be better understood in coupled models. A comparison can be drawn with the simulation of cloud processes where the physics occurs on scales smaller than are modelled. Progress can be made using earth system models that integrate more feedbacks and through the evolution to ever-higher resolution. A convergence of these issues was highlighted by Swingedouw *et al.* [58], who found that increased resolution around cryospheric and meltwater processes resulted in far more immediate connection between Greenland ice melt and AMOC stability, raising the question of whether the CMIP6 models can successfully reproduce potentially abrupt changes in the AMOC system. The potential catastrophe of such a scenario is put in sharp contrast when statistical observational estimates of the approach of an AMOC tipping point has already been indicated [59].

## 7. Conclusion

It is said that the only person who believes a model is the person who built it and that everyone believes an experiment/observation apart from the person who performed it. The challenge facing the AMOC community is either to reconcile the differences between climate models and observations or to better understand the reasons for deviation. Only with improved agreement and convergence will future IPCC reports be able to move from assessments of *low confidence* in this crucial climate variable.

We finish with a pessimistic statement: if it is not possible to reconcile climate models and observations of the AMOC in the historical period, then we believe the statements about future confidence about AMOC evolution should be revised. Low confidence in the past should mean lower confidence for the future! The IPCC AR6 report ranks it as *very likely* that the AMOC will decline in a changing climate. But, if these models cannot reproduce past variations, why should we be so confident about their ability to predict the future? Likewise, the IPCC AR6 reports medium confidence that there will be no collapse in the AMOC. But, with missing ice and freshwater dynamics, the processes that could initiate a collapse are unlikely to be simulated. We believe that progress needs to be made in understanding why models do not reproduce past AMOC variability and that this is the key to having confidence in the future evolution of this key climate variable.

## Data Availability

Data used in this manuscript is reproduced from previous publications. Each specific publication has been referenced. Access to each dataset can be found in those original references or by contacting the original authors. No new data were produced for this paper.
